# Myeloid-derived suppressor cells in colorectal cancer: mechanisms of immunosuppression, therapy resistance and therapeutic targeting

**DOI:** 10.3389/fcell.2026.1840613

**Published:** 2026-06-26

**Authors:** Haoyi Zhang, Wenyue Liu, Yutong Zhang, Shuchen Chen, Gongping Sun

**Affiliations:** 1 Department of General Surgery, The Fourth Affiliated Hospital, China Medical University, Shenyang, China; 2 Department of Lung Cancer Center, Liaoning Cancer Hospital & Institute, Cancer Hospital of Dalian University of Technology, Cancer Hospital of China Medical University, Shenyang, China

**Keywords:** colorectal cancer, immune checkpoint blockade, immunotherapy resistance, myeloid-derived suppressor cells, tumor microenvironment

## Abstract

The overall response of colorectal cancer (CRC) to immune checkpoint blockade remains limited, particularly in patients with microsatellite-stable disease. One important underlying mechanism is the involvement of myeloid-derived suppressor cells (MDSCs) in shaping an immunosuppressive TME. Under the influence of tumor-associated genetic alterations, chronic inflammation, the intestinal microbiota, metabolic stress, and therapeutic pressure, MDSCs undergo aberrant expansion and functional skewing. By remodeling the local immune ecology, they attenuate T cell- and natural killer (NK) cell-mediated antitumor responses. Concurrently, MDSCs are also implicated in angiogenesis, barrier disruption, stromal remodeling, premetastatic niche formation, and therapeutic tolerance. Thus, MDSCs are not only critical mediators of immune evasion but also key components of CRC progression and treatment resistance. Current clinical translation in this field remains constrained by the ambiguous definition of human MDSCs, phenotypic overlap, insufficient functional validation, and imprecise patient stratification. Future studies should integrate single-cell omics, spatial omics, metabolic profiling, and microbiome analyses to establish more functionally oriented biomarkers. On this basis, combination therapeutic strategies targeting MDSC recruitment, suppressive function, or reprogramming states should be further developed.

## 1 Introduction

CRC remains one of the leading causes of cancer-related morbidity and mortality worldwide, with therapeutic failure largely attributable to metastatic dissemination and the limited durability of systemic treatments (Bray et al., 2024). Although immune checkpoint blockade (ICB) has reshaped the therapeutic landscape of multiple malignancies, its clinical benefit in CRC remains highly uneven (Jia et al., 2024). Patients with tumors characterized by high microsatellite instability or deficient mismatch repair may achieve meaningful therapeutic responses, whereas the majority of patients with microsatellite-stable (MSS) CRC derive minimal benefit from ICB monotherapy (Zhang et al., 2026). This clinical disparity underscores a central unresolved question in CRC immunology: despite the presence of tumor antigens and the expanding availability of immune-based therapies, why do most CRCs remain immunologically “cold” or immune-excluded? Accumulating evidence indicates that this resistance cannot be explained solely by impaired T-cell priming or aberrant expression of checkpoint ligands. Rather, it reflects a multilayered suppressive TME, in which pathological myeloid cells, stromal barriers, metabolic constraints, and microbiota-associated inflammation act in concert to restrict effective antitumor immunity (Centeno-Girona et al., 2026). The therapeutic challenge of MSS CRC cannot be attributed merely to a lower mutational burden (Chen et al., 2025b). In many cases, even in the presence of tumor-associated antigens, sustained and effective antitumor immunity fails to develop. Inflammatory cell infiltration is frequently observed in pathological sections; however, this does not necessarily equate to effective immune-mediated cytotoxicity (Koh et al., 2026). In some tumors, the invasive margin appears immunologically active, whereas the tumor core lacks lymphocytes capable of directly engaging cancer cells and exerting effector functions (Wankhede et al., 2025). In recent years, evidence from human specimens, multi-omics analyses, and clinical cohort studies has gradually converged on a shared understanding. The low responsiveness of CRC to immunotherapy is often not caused by the failure of a single molecular target (Han et al., 2025a). Impaired immune-cell infiltration constitutes an important foundation. The local suppressive immune ecosystem further attenuates immune effector activity. Abnormal tissue architecture also limits physical contact between immune cells and tumor cells. Treatment-induced adaptive alterations may, in turn, sustain a state of low responsiveness. These factors are superimposed and collectively shape this therapeutic dilemma.

In this context, myeloid cells have re-emerged as a major focus in CRC immunology (van Vlerken-Ysla et al., 2023). For a considerable period, tumor immunology predominantly centered on T-cell recognition, activation, and exhaustion, whereas myeloid cells were often regarded as a concomitant manifestation of inflammation (Nakamura and Smyth, 2020; Anshu et al., 2026). This view now appears overly simplistic. During sustained tumor growth, the host hematopoietic system remains under chronic stress (Karin, 2020). Consequently, a spectrum of incompletely matured myeloid cells with functionally skewed phenotypes emerges in the peripheral blood and within the tumor milieu (Bronte et al., 2016; Veglia et al., 2021). These cells retain certain features of innate immunity while acquiring pathological functions that support tumor survival (Tcyganov et al., 2018). MDSCs were proposed within this conceptual framework and have been continually refined in subsequent studies (Gabrilovich et al., 2007). Unlike CD8^+^ T cells, MDSCs do not possess clearly demarcated lineage boundaries (Bronte et al., 2016). More precisely, they represent a functional assemblage of tumor-associated myeloid cells generated under specific pathological conditions.

CRC is characterized by a distinctive environmental context (Kayama et al., 2020; Kang et al., 2025). The intestine is chronically exposed to dietary components, bile acids, commensal bacteria, and their metabolites (Filardy et al., 2023; Kayama et al., 2020; Glotfelty et al., 2020; Monte et al., 2025). Accordingly, the mucosal immune system must maintain a finely regulated balance between tolerance and host defense (Schären and Hapfelmeier, 2021). Once tumorigenesis occurs, this equilibrium is disrupted (Peddireddi et al., 2025). Epithelial renewal subsequently becomes dysregulated (Hada and Xiao, 2025). Local inflammation persists, and barrier function progressively declines (Jensen et al., 2022). As a consequence, microbiota-associated signals gain easier access to deeper tissue compartments (Kouzu et al., 2022; Mutignani et al., 2021). Concurrently, genetic alterations intrinsic to cancer cells also contribute to this process (Wu et al., 2024; Chen et al., 2025a). These alterations further modify the manner in which surrounding tissues recruit immune cells (He et al., 2025). Myeloid dysregulation in CRC is not merely a secondary consequence of increasing tumor burden. Rather, it is more likely to result from the interplay among intestinal organ-specific features, tumor evolution, and host responses (Zhang et al., 2026). Precisely for this reason, the biological implications of cells collectively referred to as MDSCs are not fixed. In peripheral blood, they may reflect systemic inflammation and hematopoietic stress. In primary lesions, they may participate in local immunosuppression. In liver metastases, they may be reshaped by the TME of the metastatic organ (Hu et al., 2026a; Zeng et al., 2021a). In residual lesions after treatment, they may further represent an adaptive survival state (Li et al., 2025d).

MDSCs provide an important entry point for understanding immune resistance in CRC. They link tumor cell-intrinsic alterations, the intestinal microecology, chronic inflammation, tissue architecture, and post-treatment adaptation. At the same time, they also reveal several limitations in current research, including the lack of a unified definition, the limited translational extrapolability of experimental models, insufficient functional validation, and relatively crude patient stratification. This review discusses the developmental context, functional differentiation, microenvironmental roles, and therapeutic translation of this cellular population in colorectal cancer. Particular emphasis is placed on the concept that MDSCs do not represent a fixed cellular lineage but rather a dynamic pathological state. By synthesizing the existing evidence, this review aims to provide a basis for the subsequent establishment of more reliable biomarkers and more precise combination therapeutic strategies.

## 2 MDSC landscape in colorectal cancer

### 2.1 Pathological myelopoiesis and recruitment

Under normal physiological conditions, bone marrow progenitor cells differentiate into mature myeloid cells with functions in immune defense and tissue repair (Weiskirchen et al., 2019; Fresno and Gironès, 2021). However, during the initiation and progression of CRC, multiple chemotactic and inflammatory factors are continuously released, thereby disrupting the homeostatic differentiation trajectory of myeloid cells (Nakamura and Smyth, 2020; Wang et al., 2025; Frascatani et al., 2026). This ultimately induces the generation of “stress-associated” or “pathological” myeloid cells (Silva-Romeiro et al., 2025; Hu et al., 2025). During this process, extensive aberrant expansion of immature myeloid cells provides a foundation for tumor immune evasion (Dash et al., 2026).

Specifically, CRC tissues and surrounding stromal cells continuously secrete factors such as interleukins, colony-stimulating factors, angiogenic factors, and prostaglandins (Eruslanov et al., 2010; Liao et al., 2025; Chun et al., 2015). These factors not only induce persistent local inflammation but also drive aberrant expansion of myeloid progenitor cells through feedback loops. Concurrently, remodeling of the TME further promotes acquisition of more pronounced immunosuppressive features by newly generated cells, thereby exacerbating the suppression of lymphocyte function (Xu et al., 2024; Liao et al., 2019). In addition, CRC is frequently accompanied by Kirsten rat sarcoma viral oncogene homolog (KRAS) mutations or dysregulation of the Wnt/β-catenin signaling pathway (Zhou et al., 2023; Dang et al., 2022; Zhai et al., 2023; Chen et al., 2019). These tumor-intrinsic alterations can likewise interfere with aberrant differentiation of myeloid cells and promote enrichment of polymorphonuclear myeloid-derived suppressor cells (PMN-MDSCs) or monocytic myeloid-derived suppressor cells (M-MDSCs) within the local microenvironment.

At the level of recruitment mechanisms, multiple chemokine axes collectively constitute the regulatory network governing the enrichment of MDSCs in CRC (Hu et al., 2026b; Zhang et al., 2025a; Shi et al., 2019). C-X-C motif chemokine ligand (CXCL) family members and their receptor C-X-C motif chemokine receptor 2 (CXCR2) primarily promote the mobilization and migration of polymorphonuclear MDSCs (PMN-MDSCs) (Gao et al., 2025; Yang et al., 2024a). C-C motif chemokine ligand (CCL) family members and C-C motif chemokine receptor 2 (CCR2) recruit a greater number of monocytic MDSCs (M-MDSCs). Molecules such as S100 proteins (Guo et al., 2025b; Özbay Kurt et al., 2024), complement component 5a (C5a) (Yang et al., 2025), and stem cell factor (Pan et al., 2008) may act similarly with prostaglandin signaling (Mamand et al., 2024) to participate in the localization and long-term retention of myeloid cells at tumor sites. These pathways exhibit marked functional redundancy (Shi et al., 2019). When a key node is inhibited, tumors may exploit alternative pathways to sustain the continuous accumulation of MDSCs. Strategies targeting a single chemokine axis have shown evident benefit only in specific models, whereas their efficacy remains limited in complex clinical settings (Yang et al., 2024a).

**FIGURE 1:**
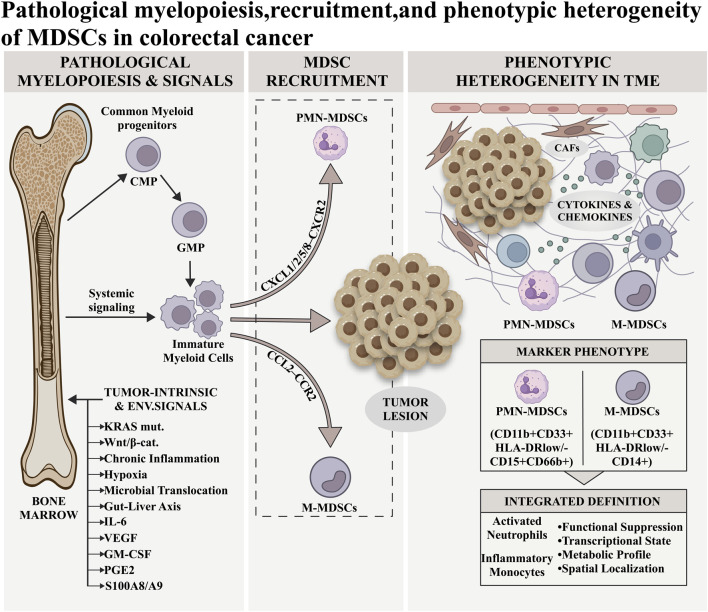
Pathological myelopoiesis, recruitment, and phenotypic heterogeneity of MDSCs in colorectal cancer. Colorectal cancer reshapes systemic and local myelopoiesis through persistent inflammatory, metabolic, microbial, and tumor-intrinsic signals. Tumor cells, cancer-associated fibroblasts, endothelial cells, and inflammatory immune cells release cytokines, chemokines, growth factors, prostaglandins, complement components, and S100 family proteins, thereby driving the expansion of immature myeloid populations and their recruitment into tumor lesions. CXCL–CXCR2 signaling predominantly promotes the mobilization and infiltration of PMN-MDSCs, whereas CCL2–CCR2 signaling is more closely associated with M-MDSC recruitment. Additional pathways, including C5a, S100A8/A9, stem cell factor, VEGF, GM-CSF, IL-6, and PGE2, support MDSC retention and functional polarization in the tumor microenvironment. In colorectal cancer, KRAS mutation, Wnt/β-catenin activation, chronic mucosal inflammation, microbial translocation, hypoxia, and gut–liver axis signaling further amplify pathological myeloid remodeling. Human MDSCs are commonly enriched using HLA-DR^low^/− and myeloid markers such as CD11b and CD33, with CD14, CD15, and CD66b used to distinguish monocytic and granulocytic subsets. However, phenotypic overlap with activated neutrophils, low-density neutrophils, and inflammatory monocytes limits marker-based classification. Therefore, functional suppression, transcriptional state, metabolic profile, and spatial localization should be integrated to define clinically meaningful MDSC states in colorectal cancer.

Compared with other tissues, the distinctive microenvironmental features of the intestine provide more complex determinants of myeloid immune regulation in CRC. The intestinal mucosa is chronically exposed to diverse external stimuli, which may induce bone marrow to release large numbers of immature myeloid cells (Karin, 2020). After these cells accumulate in tumor regions with pronounced invasiveness or metabolic imbalance, they may amplify immunosuppressive networks through multiple signaling pathways (Ashkenazi-Preiser et al., 2024; Zhou et al., 2025a; Guo et al., 2025a; Xun et al., 2026; Xie et al., 2025). The intestinal-hepatic axis in CRC may also influence the liver by remodeling the hepatic immune microenvironment before the occurrence of liver metastasis, thereby facilitating tumor colonization and growth (Li et al., 2025a; Zeng et al., 2021b). The coordinated interaction between the intestinal microenvironment and distal organs jointly influences the distribution of MDSCs in CRC (Ashkenazi-Preiser et al., 2024; Xun et al., 2026). However, current understanding of the microbiota is still derived primarily from murine or *in vitro* models. Direct evidence from *in vivo* experiments and clinical studies remains insufficient. Future studies integrating large-scale multi-omics approaches with spatial localization technologies may further elucidate *in vivo* activation mechanisms of microbiota-associated MDSCs (Figure 1).

### 2.2 MDSC heterogeneity and definition

The heterogeneity of MDSCs remains a major challenge in the field of CRC immunology (Bronte et al., 2016). MDSCs can be classified into three major subsets: polymorphonuclear MDSCs (PMN-MDSCs), monocytic MDSCs (M-MDSCs), and immature MDSCs. In murine models, PMN-MDSCs are typically characterized by high *Ly6G* expression and intermediate *Ly6C* expression, whereas M-MDSCs may exhibit high *Ly6C* expression but low *Ly6G* expression. However, the situation is considerably more complex for human MDSCs. The inflammatory milieu, sampling site, detection method, and interindividual variability are all important influencing factors. In addition, human myeloid cells display a phenotypic continuum. These factors make the classification and identification of human MDSCs particularly challenging (Table 1).

**TABLE 1:** Phenotypic and functional comparison of murine and human MDSCs. This table summarizes the major subsets, commonly used surface markers, functional suppressive mechanisms, and tissue-context considerations for murine and human myeloid-derived suppressor cells. It highlights the differences between mouse and human MDSC identification and emphasizes the need for functional validation beyond marker-based classification.

Feature	Murine MDSCs	Human MDSCs
Major subsets	Murine MDSCs are commonly divided into PMN-MDSCs, M-MDSCs and immature MDSCs	Human MDSCs are also classified as PMN-MDSCs, M-MDSCs and early-stage or immature MDSCs
General myeloid phenotype	CD11b^+^Gr-1^+^ cells are commonly used as the broad MDSC population in mice	CD11b^+^CD33^+^HLA-DR^low^/− cells are frequently used for initial enrichment of human MDSCs
PMN-MDSC phenotype	CD11b^+^Ly6G^+^Ly6C^low^/int	CD11b^+^CD33^+^HLA-DR^low^/−CD15^+^ or CD66b^+^CD14^−^
M-MDSC phenotype	CD11b^+^Ly6G^−^Ly6C^high^	CD11b^+^CD33^+^HLA-DR^low^/−CD14^+^CD15^−^
Immature or early-stage MDSCs	Less uniformly defined; often included within immature CD11b^+^Gr-1^+^ myeloid populations	Lin^−^HLA-DR^low^/−CD33^+^ cells or CD33^+^ cells lacking mature lineage markers
Functional suppressive mechanisms	ARG1, iNOS, ROS, RNS, NO and STAT3-associated programs suppress T-cell responses	ARG1, iNOS, ROS/RNS, adenosine signaling, STAT3 activation and impaired antigen presentation contribute to immune suppression
Spatial and tissue context	Murine models allow mechanistic interrogation of MDSC recruitment, expansion and function	Human MDSCs show strong heterogeneity according to sampling site, tumor region, inflammation and treatment status

In clinical studies, MDSCs are usually preliminarily enriched on the basis of low human leukocyte antigen DR (*HLA-DR*) expression in combination with myeloid markers such as *CD11b* and *CD33*. Monocytic and granulocytic subsets are then distinguished according to the expression of molecules including *CD14*, *CD15*, and *CD66b*. Immature MDSCs are characterized by low *HLA-DR* expression and negativity for lineage markers, or by *CD33* positivity without terminal differentiation. Reliance solely on these surface markers cannot accurately define MDSC subsets. Activated neutrophils, low-density neutrophils, and inflammatory monocytes may all phenotypically overlap with MDSCs, and their boundaries are not clearly demarcated. More importantly, phenotypic similarity does not necessarily indicate functional equivalence. Some activated neutrophils without immunosuppressive activity are frequently misclassified as MDSCs. Conclusions derived from such analyses may therefore be affected, thereby weakening the reliability of subsequent translational studies. When evaluating MDSCs in CRC, key information regarding their immunosuppressive capacity, transcriptional dynamics, metabolic profiles, and anatomical localization should also be integrated (Li et al., 2026). At the functional level, particular attention should be given to their immunosuppressive activity, thereby enabling a more accurate assessment of the actual suppressive potential of specific myeloid populations. In addition, myeloid cells exhibit distinct spatial distribution patterns. In primary and metastatic lesions, the spatial localization, local signaling milieu, and cellular neighborhood relationships of MDSC subsets may all influence their roles in immune evasion (Ding et al., 2025). Recent single-cell transcriptomic and spatial omics studies have shown that MDSCs in human CRC do not fully correspond to conventional classifications. Instead, they exist as a continuum of cellular states with varying degrees of activation and functional plasticity (Li et al., 2026). Among suppressive cells involved in cancer progression, certain cellular subsets are frequently associated with poor prognosis (Wang et al., 2026a). Other subsets may exacerbate local inflammatory responses and restrict CD8^+^ T-cell function (Han et al., 2025b). Their suppressive functions still require confirmation through both functional assays and spatial validation.

Therefore, classification of MDSCs solely as “granulocytic” or “monocytic” populations may obscure their functional heterogeneity. Their definition should not depend exclusively on surface phenotypes. Only multidimensional assessment can determine whether a specific subset genuinely exerts immunosuppressive functions. Precise delineation of MDSC subsets has important research and clinical significance. It facilitates a more accurate evaluation of the effects of different subsets on the CRC immune microenvironment and helps clarify their association with clinical therapeutic efficacy.

### 2.3 Spatial organization of MDSCs

At the spatial and functional levels, the role of MDSCs in CRC is not determined solely by their abundance. Their local positioning is equally critical (Marteau et al., 2026; Li et al., 2026). The CRC TME can be regarded as a compartmentalized ecosystem in which malignant epithelial cells, stromal components, immune cells, vascular networks, extracellular matrix, and metabolites interact to generate distinct tissue compartments. MDSCs are not uniformly distributed within tumors. Rather, they accumulate at specific sites and exert immunosuppressive functions within these local niches.

PMN-MDSCs are not randomly distributed within tumor tissues. They are typically enriched at the invasive margin, in hypoxic and necrotic regions, and within desmoplastic stromal areas (He et al., 2025; Marteau et al., 2026). These regions often share several common features, including elevated lactate levels, activation of hypoxia-inducible factor 1α (*HIF-1α*), persistent chemokine release, and continuous accumulation of extracellular matrix degradation fragments (Corzo et al., 2010). The convergence of these signals enhances the immunosuppressive capacity of PMN-MDSCs. Distinct spatial locations also confer different functions on PMN-MDSCs. At the invasive front, PMN-MDSCs may release reactive oxygen species and nitric oxide. They may also induce the formation of neutrophil extracellular traps and secrete matrix-degrading enzymes. These functions restrict the entry of effector lymphocytes into tumor tissues (Zhang et al., 2025a). In hypoxic regions, PMN-MDSCs activate hypoxia-inducible factor 1α (*HIF-1α*)-associated signaling. Adenosine synthesis and angiogenic factors are subsequently upregulated. Thus, PMN-MDSCs not only reinforce local immunosuppression but also participate in tissue remodeling and angiogenesis.

The spatial distribution of M-MDSCs is more strongly biased toward tumor nests, perivascular regions, and macrophage-enriched zones (Gao et al., 2024; Cheng et al., 2026). This localization facilitates their contact with malignant cells and tumor-associated macrophages. Accordingly, M-MDSCs exhibit more pronounced bidirectional plasticity (Galdiero et al., 2013). Under the influence of local cytokines and metabolic signals, M-MDSCs may maintain a monocytic suppressive phenotype or further differentiate into macrophage-like subsets (Liu et al., 2026a; Li et al., 2025c). Under specific reprogramming conditions, a proportion of these cells may even restore a certain degree of antigen-presenting capacity (Zhang et al., 2025c). Their ultimate fate is therefore not fixed. Multiple microenvironmental signals collectively shape the functional trajectory of M-MDSCs, enabling their dynamic transition between immune tolerance and inflammatory responses (Wang et al., 2024b).

MDSCs also participate in the formation of immune-excluded tumor architecture. They do not act in isolation; rather, MDSCs continuously interact with fibroblasts, endothelial cells, regulatory T cells, and tumor-associated macrophages, thereby forming a dense stromal microenvironment. This structure restricts the entry of CD8^+^ T cells into the tumor core (Iwane et al., 2025). MDSCs release multiple inflammatory and pro-angiogenic signals that activate fibroblasts and endothelial cells (Galdiero et al., 2013). Stromal cells further remodel extracellular matrix and enhance chemotactic signaling. As a result, suppressive myeloid cells are persistently retained within stromal compartments. Myeloid accumulation, stromal activation, and aberrant angiogenesis influence one another. This feature is particularly prominent in MSS CRC, in which tumors are more prone to forming an immune-excluded microenvironment. This spatial immune remodeling is not confined to primary lesions. When CRC undergoes distant metastasis, the spatial distribution of MDSCs remains a key determinant of immune remodeling within metastatic lesions (Liu et al., 2026a). Because of its distinctive architecture, the liver continuously receives microbial components and metabolite-derived signals from the portal circulation (Ralli et al., 2023; Dey, 2025). Before extensive dissemination of tumor cells, premetastatic signals released from the primary lesion through the gut-liver axis may reshape the intrahepatic immune ecosystem in advance (Lan et al., 2026; Zeng et al., 2021a; Hu et al., 2026a).

**FIGURE 2:**
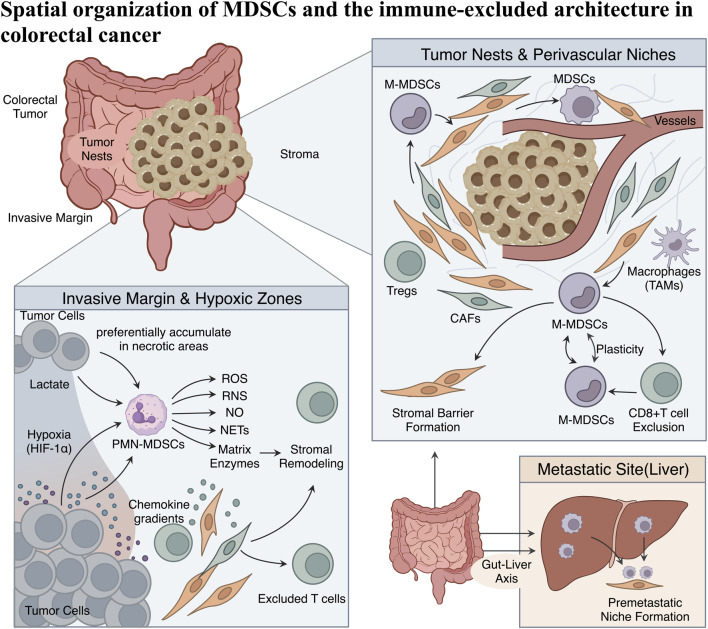
Spatial organization of MDSCs and immune-excluded architecture in colorectal cancer. The biological impact of MDSCs in colorectal cancer is determined not only by their abundance but also by their anatomical localization within distinct tumor compartments. PMN-MDSCs preferentially accumulate at the invasive margin, hypoxic and necrotic regions, and desmoplastic stromal areas, where lactate accumulation, HIF-1α activation, chemokine gradients, extracellular matrix degradation products, and inflammatory mediators enhance their suppressive and tissue-remodeling functions. In these niches, PMN-MDSCs produce reactive oxygen species, reactive nitrogen species, nitric oxide, neutrophil extracellular traps, matrix-degrading enzymes, and pro-angiogenic factors, thereby restricting effector lymphocyte infiltration and promoting stromal remodeling. M-MDSCs are more frequently located around tumor nests, perivascular regions, and macrophage-enriched areas. Their close contact with malignant epithelial cells, tumor-associated macrophages, endothelial cells, and stromal cells enables dynamic plasticity, allowing transition between monocytic suppressive states, macrophage-like phenotypes, or partially reprogrammed antigen-presenting states. Through reciprocal interactions with CAFs, Tregs, TAMs, endothelial cells, and extracellular matrix components, MDSCs contribute to the formation of a dense stromal barrier that excludes CD8^+^ T cells from the tumor core. This spatial immune remodeling is particularly relevant in microsatellite-stable colorectal cancer and may extend to metastatic sites, especially the liver, where gut–liver axis-derived microbial and metabolic signals support premetastatic niche formation.

Current spatial studies of MDSCs remain markedly insufficient. Many studies focus only on the overall abundance of MDSCs, whereas the spatial relationships between MDSCs and the tumor microenvironment in which they reside remain inadequately characterized. Understanding of the dynamic changes in MDSCs is likewise limited, and their spatiotemporal evolutionary patterns remain unclear. The criteria used to define MDSC spatial localization are also inconsistent. Future studies may integrate spatial omics technologies to further investigate the association between the spatial positioning of MDSCs and their immunosuppressive function (The core mechanisms described in this section are summarized in Figure 2).

## 3 MDSCs in CRC progression and resistance

### 3.1 Metabolic immune suppression

MDSCs can modulate immunity in CRC by interfering with metabolic processes, representing an important mechanism by which they restrict antitumor immunity. Within the TME, effector T cells and NK cells require relatively stable metabolic conditions (Li et al., 2025b). Adequate nutrient availability influences their proliferative capacity, whereas balanced redox homeostasis affects their activation status (Cai et al., 2025; Yang and Wang, 2025). When energy metabolism is dysregulated, the immune surveillance and cytotoxic functions of effector T cells decline. MDSCs exert suppressive effects precisely by disrupting these conditions (Liu et al., 2026b). They can deplete key amino acids such as L-arginine, making it difficult for T cells to maintain normal proliferation (Ishitobi et al., 2022; Grzywa et al., 2020). Their effector functions also become more prone to exhaustion. In parallel, MDSCs can generate large amounts of reactive oxygen species (ROS) and reactive nitrogen species (RNS) (Bergerud et al., 2024). These molecules interfere with T-cell receptor signaling, thereby limiting T-cell activation, migration, and target recognition. In addition, nitric oxide (NO) production mediated by inducible nitric oxide synthase (*iNOS*) is also involved in this process. Excessive nitric oxide can impair T-cell function and affect antigen presentation by dendritic cells (Liu et al., 2024; Ke et al., 2020; Cartwright et al., 2021; Markowitz et al., 2017). The convergence of multiple metabolic disturbances ultimately attenuates antitumor immune responses in CRC.

The intestinal microenvironment of CRC is also characterized by hypoxia, acid accumulation, and elevated lactate levels. These metabolic alterations further influence the recruitment and functional state of MDSCs. Such distinctive features of the intestinal microenvironment can activate *HIF-1α*-associated pathways, thereby rendering MDSCs more prone to transition toward an immunosuppressive phenotype (Noman et al., 2014). Meanwhile, the expression of molecules such as *CD39* and *CD73* is upregulated, promoting the conversion of extracellular adenosine triphosphate (ATP) into adenosine (Kim et al., 2021; Hajizadeh et al., 2020; Kumar, 2013). Once accumulated, adenosine exerts suppressive effects through A2A or A2B receptors. The cytotoxic activity of T cells and NK cells subsequently declines, and their immune clearance capacity is concomitantly weakened. Under these conditions, MDSCs not only suppress effector lymphocyte function but also participate in competition for nutritional and metabolic resources. The availability of oxygen, glucose, and amino acids is further reduced, ultimately leading to persistent attenuation of antitumor immunity in CRC.

Beyond canonical metabolic pathways, epitranscriptomic modifications, such as N6-methyladenosine (*m6A*), may also participate in shaping the function of MDSCs (Feng et al., 2024; Li et al., 2025c; Wang et al., 2023a). The *m6A* regulatory system can influence immune-related messenger RNA (mRNA). Inflammatory responses, chemotactic signaling, and immune checkpoint expression may all be affected. In myeloid cells, this regulation may further influence MDSC expansion and activation, with consequent alterations in the functional state of MDSCs. At a deeper level, epitranscriptomic modifications may serve as a link between metabolic reprogramming and immunosuppression (Wu et al., 2024; Fawzul Ameer et al., 2026). Tumor burden, hypoxia, inflammatory stimulation, and metabolic perturbations collectively constitute external pressures. Under these pressures, MDSCs are not fixed entities. Rather, they more closely resemble a plastic myeloid state (Kanterman et al., 2014; Deng et al., 2019; Gneo et al., 2022; Leonard et al., 2016). Their metabolic profiles may adjust in response to microenvironmental changes, accompanied by concomitant alterations in epitranscriptomic features.

### 3.2 Cellular crosstalk

Within the CRC TME, MDSCs do not act in isolation. They are closely interconnected with multiple non-malignant cellular components (Hu et al., 2026a; Gallo et al., 2021; Tang et al., 2020). Among these cellular constituents, cancer-associated fibroblasts (CAFs) are intimately linked to MDSCs. CAFs can secrete inflammatory mediators and participate in extracellular matrix remodeling. They also alter the mechanical properties of local tissues, leading to the formation of a dense stromal architecture (Benedicto et al., 2018; Gallo et al., 2021; Wang et al., 2022). This ultimately restricts lymphocyte infiltration into the tumor core. Conversely, MDSCs can also shape the state of CAFs (Li et al., 2025a). The growth factors, proteases, and immunomodulatory molecules released by MDSCs promote CAF activation (Sieminska and Baran, 2020; Legitimo et al., 2014; OuYang et al., 2015; Grauers Wiktorin et al., 2019; Ye et al., 2017; Kobayashi et al., 2022; Miyashita and Saito, 2021; Li et al., 2024a; Bai et al., 2015; Drebert et al., 2018; Ko et al., 2000). Myeloid cells and stromal components thereby reinforce one another, making it more difficult for T cells to enter malignant epithelial regions.

MDSCs may also act synergistically with regulatory T cells (Tregs), jointly maintaining local immune tolerance (Gabrilovich and Nagaraj, 2009). This cooperation does not depend on a single pathway. MDSCs can release suppressive signals and deplete key metabolic substrates. They also inhibit dendritic cell activation and impair antigen presentation. Under these influences, Tregs are more likely to expand, while the priming responses of effector T cells are concomitantly weakened (Silva-Romeiro et al., 2025). Tregs can likewise exert reciprocal effects on MDSCs. The immunomodulatory factors released by Tregs may further stabilize the suppressive phenotype of MDSCs. This relationship more readily blunts immune responses. Although inflammatory cell infiltration is commonly observed in CRC lesions, inflammatory infiltration is not equivalent to effective antitumor immunity (Cives-Losada et al., 2026). If MDSCs and Tregs predominate locally, cytotoxic immune responses may still be suppressed. Under these circumstances, even when inflammatory signals persist, effector T cells may be unable to exert sufficient function.

Beyond the interaction between MDSCs and Tregs, reciprocal shaping within the myeloid compartment also influences the CRC immune microenvironment (Centeno-Girona et al., 2026; Huang and Fang, 2025). Within the TME, M-MDSCs continuously receive differentiation cues from tumor cells, stromal cells, and immune cells under conditions such as hypoxia, inflammatory stimulation, and dysregulated lipid metabolism (Frascatani et al., 2026; Ke et al., 2020; Ibrahim et al., 2020; Wang et al., 2020b). Their functional state is consequently altered. A subset of M-MDSCs may gradually transition toward an immunosuppressive macrophage-like phenotype. As a result, T-cell responses are more strongly restricted, and antigen-presenting function may be further diminished. Hypoxic signaling plays an important role in this process. It can activate *HIF-1α*-associated pathways and drive phenotypic skewing of myeloid cells (Qi et al., 2022; Yang et al., 2024b; Wang et al., 2026b; Deng et al., 2019; Corzo et al., 2010). Meanwhile, reactive oxygen species, nitric oxide, and other metabolites accumulated within tumor tissues may also impair the function of surrounding immune cells. Tumor-associated macrophages (TAMs) are not passive recipients of signals within this network. They can release chemotactic and immunoregulatory signals that promote the recruitment, retention, and functional maintenance of M-MDSCs (Yang et al., 2024a; Long et al., 2019; Hu et al., 2026a). MDSCs can also reciprocally modulate the state of TAMs, thereby shifting the overall myeloid cell population toward a more immunosuppressive phenotype. As this communication is progressively reinforced, T-cell activity is further attenuated, ultimately contributing to tumor progression.

### 3.3 Angiogenesis and vascular remodeling

Beyond immunosuppression, MDSCs may also participate in the remodeling of the vascular ecology in CRC. Their effects are not confined to the regulation of immune cells. MDSCs can also directly influence endothelial cells, stromal architecture, and local material exchange (Blidner et al., 2025; Tartour et al., 2011). For rapidly progressing tumors, blood vessels are not merely conduits for nutrient delivery. They determine the supply of oxygen and nutrients and influence the clearance of metabolic waste (Chen and Zhou, 2025; Liu et al., 2023). In addition, vascular architecture affects the efficiency with which immune cells enter tumor tissues and provides potential routes for tumor cell extravasation. MDSCs can continuously release pro-angiogenic signals. Vascular endothelial growth factor (*VEGF*), basic fibroblast growth factor (*bFGF*), and angiopoietins are representative mediators (Tartour et al., 2011; Mackert et al., 2017; Liu et al., 2023). After acting on endothelial cells, these signals promote endothelial proliferation and migration. The capacity of endothelial cells to form nascent lumens is also enhanced (Blidner et al., 2025; Liu et al., 2023). Endothelial cells that were originally relatively quiescent gradually shift toward an activated state, accompanied by an increase in neovascularization. This process is particularly prominent in tumor regions characterized by marked hypoxia and metabolic stress. Hypoxic signaling can enhance the activity of *VEGF*-associated pathways. Endothelial cell activity is further increased, and microvascular permeability is also elevated. Under these conditions, the CRC vascular network gradually exhibits abnormal dilation and structural remodeling (Mo et al., 2025; Zong et al., 2017; Sieminska and Baran, 2020; Rahma and Hodi, 2019). After vascular permeability increases, material exchange within the local CRC microenvironment is also altered. Oxygen, glucose, and amino acids more readily enter tumor tissues, providing tumor cells with the substrates required for sustained proliferation. Metabolic waste, inflammatory mediators, and necrosis-associated products are also redistributed locally, further increasing microenvironmental complexity (Bahrami et al., 2024; Sieminska and Baran, 2020). This alteration does not indicate normalization of vascular function. In contrast, tumor-associated neovessels are often structurally disorganized and functionally unstable (Battaglin et al., 2018; Lopez et al., 2019). Their luminal morphology is irregular, and blood flow distribution is uneven. Some regions receive relatively sufficient perfusion, whereas others remain hypoxic. Thus, spatial heterogeneity within CRC is pronounced. As MDSC abundance increases or their activation is enhanced, pro-angiogenic signals continue to accumulate, rendering the vascular network increasingly dense (Chen et al., 2021; Xu et al., 2018; Mackert et al., 2017). For tumors, this aberrant blood supply has dual effects. On the one hand, it provides support for growth; on the other hand, it creates conditions conducive to immune evasion and local invasion.

The effects of MDSCs on angiogenesis are also reflected in extracellular matrix remodeling. Stromal deposition is commonly observed in CRC lesions, rendering local tissues more compact. This structural alteration can restrict vascular extension into deeper tumor regions and modify the routes of cellular migration. As tumors expand, the local stroma undergoes loosening. Matrix metalloproteinases secreted by MDSCs may participate in this process (Wu et al., 2023; Itatani et al., 2013; Gan et al., 2019). Enzymes represented by matrix metalloproteinase 9 (*MMP-9*) can degrade collagen, elastin, and other matrix components (Herszényi et al., 2012; Liang et al., 2020). As a result, the originally robust mechanical barrier is weakened. Pro-angiogenic signals embedded within the extracellular matrix are re-released. Endothelial cells acquire greater migratory space, and nascent vessels can more readily extend into deeper tumor regions. Thus, MDSCs alter local tissue architecture and create conditions conducive to angiogenesis. Signal activation and stromal loosening cooperate with each other, driving sustained vascular remodeling in CRC.

More importantly, vascular abnormalities and immunosuppression are superimposed on one another. Aberrant vasculature leads to uneven local perfusion. In some regions, insufficient blood supply aggravates hypoxia and acidification (Guo et al., 2025a). Hypoxia and an acidic milieu, in turn, promote the recruitment and activation of MDSCs. This process further impairs the function of effector T cells (Han et al., 2025b; Wang et al., 2024a). Therefore, angiogenesis is not merely a component of tumor growth; it also participates in the establishment of immune evasion. MDSCs connect vascular, metabolic, and immune processes at multiple levels. They can drive endothelial cell activation and promote expansion of the vascular network. They also participate in extracellular matrix degradation and remodeling of local barriers. These two effects cooperate to render the CRC microenvironment more permissive for sustained tumor growth. As blood flow distribution and vascular permeability are altered, tumor cells can acquire greater access to survival resources (Qi et al., 2022; Lopez et al., 2019; Mathonnet et al., 2014; Chen et al., 2021). This leads to increased interstitial pressure, tissue edema, and aggravated metabolic imbalance. These alterations continue to shape a local environment favorable for tumor dissemination (Blidner et al., 2025). Consequently, tumors are more likely to acquire a proliferative advantage and to exhibit enhanced invasive capacity and potential metastatic propensity.

### 3.4 Epithelial barrier dysfunction

Impairment of the intestinal mucosal barrier is an important feature of CRC. MDSCs are already present at the adenoma or epithelial dysplasia stage (Chun et al., 2015; Ichikawa et al., 2011; Grasso et al., 2015; Qi et al., 2025). MDSCs can be further activated by exogenous microorganisms and mucosal epithelial cells. Once activated, MDSCs exert their effects through multiple signaling pathways (Zhou et al., 2025b; Chun et al., 2015; Wang et al., 2020a; Suzuki et al., 2011). They can disrupt the stability of epithelial tight junctions and interfere with the normal operation of key molecular networks involved in mucosal repair. These alterations weaken epithelial barrier function and create conditions conducive to subsequent disease progression.

Tight junction proteins are among the first structures affected during disruption of the intestinal mucosal barrier. They maintain the fundamental integrity of the intestinal epithelium and restrict the translocation of microorganisms and toxic substances across the mucosal barrier (Landy et al., 2016; Liu et al., 2016). Tight junction proteins constitute an important defensive barrier against the dissemination of early CRC lesions. However, after MDSCs enter the intestinal mucosa, this barrier may be progressively disrupted. Within the local microenvironment, MDSCs receive inflammatory mediators and stress signals and amplify these stimuli. Subsequently, MDSCs can secrete multiple proteolytically active factors (Ichikawa et al., 2011; Wang et al., 2020a). These enzymes may degrade or modify key structural domains of tight junction proteins. Interleukin-6 (IL-6) has been shown to upregulate *claudin-2* expression and increase tight junction permeability (Suzuki et al., 2011). This leads to widening of the interepithelial spaces, loosening of the barrier architecture, and increased mucosal susceptibility. In parallel, MDSCs can indirectly promote the production of local inflammatory molecules. These signals, in turn, maintain the activated state of MDSCs. This positive-feedback pattern further exacerbates tight junction protein damage. As local permeability increases, pathogenic molecules and microbiota-derived products more readily enter the submucosa (Ahmad Kendong et al., 2021; Chen et al., 2025c). These alterations provide abnormal cells with a deeper “stealth space.” Ultimately, early malignant cells breach the mucosal barrier and progressively disseminate into surrounding tissues.

After epithelial integrity is compromised, repair programs are usually initiated by the host. Renewal of colonic mucosal stem cells and differentiation of epithelial cells can reconstruct the physiological barrier. However, the enrichment and aberrant activation of MDSCs may interfere with this process. MDSCs can release multiple factors through autocrine or paracrine mechanisms and activate related downstream proteins. These signals may disrupt epithelial cell polarity and affect mucosal renewal (Smith et al., 2020; Beury et al., 2014; Wang et al., 2026b). As MDSCs accumulate excessively at local sites, key nutrients are depleted. This impairment of repair does not arise solely from epithelial cells themselves. Rather, it essentially reflects alterations in the niche environment of colorectal epithelial stem cells. Regions of early adenoma or epithelial hyperplasia are characterized by local necrosis, hypoxia, and inflammation. Such microenvironments can promote extensive MDSC deposition and amplify their suppressive effects (Wu et al., 2024; Zhang et al., 2018; Zeng et al., 2024; Yang et al., 2024b; Gao et al., 2025; Jayakumar and Bothwell, 2017). Deposited MDSCs can release various enzymes and regulatory proteins. These molecules cooperate to establish persistent interfering signals. Once key repair signaling cascades are interrupted, the regenerative capacity of mucosal cells becomes restricted. Under the combined influence of repeated injury and chronic inflammation, the epithelial architecture gradually becomes disorganized. The barrier properties of the underlying lamina propria are also progressively weakened. Early tumor cells are thereby more likely to acquire opportunities for invasion and extend into the basal tissues.

**FIGURE 3:**
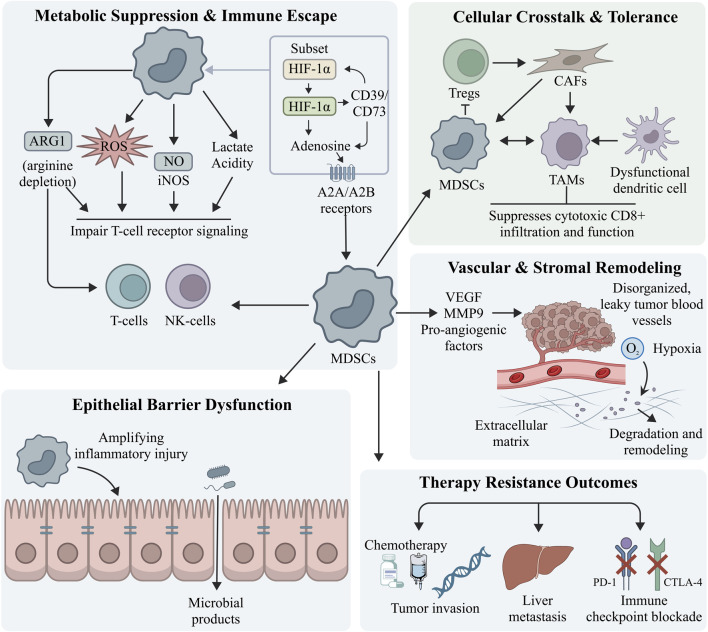
Mechanistic network by which MDSCs promote colorectal cancer progression, immune suppression, and therapy resistance. MDSCs promote colorectal cancer progression through an integrated network of metabolic, cellular, vascular, stromal, and epithelial barrier-related mechanisms. Metabolically, MDSCs deplete L-arginine through ARG1, generate reactive oxygen and nitrogen species, produce nitric oxide through iNOS, impair T-cell receptor signaling, and suppress cytotoxic T-cell and NK-cell activity. Hypoxia, lactate accumulation, acidosis, and HIF-1α activation further reinforce MDSC suppressive polarization, while CD39/CD73-mediated adenosine production inhibits antitumor immunity through A2A and A2B receptor signaling. At the cellular level, MDSCs cooperate with Tregs, CAFs, TAMs, and dysfunctional dendritic cells to maintain local immune tolerance, inhibit antigen presentation, and prevent effective CD8^+^ T-cell infiltration. MDSCs also shape vascular and stromal remodeling by releasing VEGF, bFGF, angiopoietins, MMP9, and other matrix-remodeling factors, resulting in aberrant angiogenesis, increased vascular permeability, uneven perfusion, aggravated hypoxia, and extracellular matrix degradation. In early lesions, MDSCs may contribute to epithelial barrier dysfunction by amplifying inflammatory injury, weakening tight junction integrity, interfering with mucosal repair programs, and facilitating microbial product translocation. Together, these mechanisms generate a self-reinforcing suppressive ecosystem that supports immune escape, tumor invasion, liver metastasis, and resistance to chemotherapy, anti-angiogenic therapy, radiotherapy, and immune checkpoint blockade, particularly in microsatellite-stable colorectal cancer.

These two mechanisms are complementary and mutually interactive. Together, they enable MDSCs to establish a microenvironment favorable for lesion progression as early as the initial stages of CRC. MDSCs degrade tight junction proteins, thereby rendering the intestinal epithelial barrier fragile. They also suppress mucosal repair pathways and interrupt key steps required for tissue restoration. This dual effect persistently weakens the capacity of epithelial cells to maintain homeostasis. As a result, normal mucosal architecture becomes difficult to restore. Meanwhile, neoplastic lesions may continue to progress before an effective immune response is fully initiated, thereby progressively increasing the risk of malignant deterioration (The core mechanisms described in this section are summarized in Figure 3).

## 4 Therapeutic targeting of MDSCs

### 4.1 Blocking MDSC recruitment

In CRC, the sustained recruitment of MDSCs represents an important initiating event in the formation of an immune-excluded microenvironment. This phenomenon is particularly evident in MSS CRC and CRC with liver metastasis (Zhang et al., 2025a; Umekita et al., 2024; Hu et al., 2026b). The CXCL-CXCR2 axis is mainly involved in the directed migration of granulocytic MDSCs and tumor-associated neutrophils (Gao et al., 2025). Its role is not merely an accompaniment to inflammatory responses but constitutes a critical step in driving myeloid-mediated immunosuppression. CRC tumor cells, cancer-associated fibroblasts, endothelial cells, and inflammatory myeloid cells can all secrete CXCL1, CXCL2, CXCL5, and CXCL8 (Hayashi et al., 2023; Wang et al., 2017; Qi et al., 2022; Ning et al., 2011). These chemokines form local gradients, after which myeloid cells progressively accumulate at the invasive front, in hypoxic regions, and within metastatic lesions. Relevant model studies have also shown that genetic or pharmacological inhibition of CXCR2 can markedly reduce intratumoral myeloid cell infiltration and delay intestinal tumor initiation and progression (Chen et al., 2022; Harusato et al., 2019). These findings indicate that CXCR2 signaling plays a pivotal role in myeloid cell recruitment, the establishment of immunosuppression, and tumor progression in CRC (Liao et al., 2019; Katoh et al., 2013). CXCR2 is more appropriately regarded as a key gateway regulating the myeloid immune barrier. The principal value of CXCR2 blockade does not lie in direct cytotoxicity against cancer cells but in limiting the immunosuppressive effects that arise after MDSCs enter tumor tissues. In CRC, PMN-MDSCs can act through molecules such as arginase 1 (*ARG1*), reactive oxygen species (ROS), and matrix metalloproteinase-9 (*MMP-9*). Consequently, CD8^+^ T-cell function is suppressed. Stromal degradation is further aggravated, and aberrant angiogenesis is also increased. Meanwhile, the premetastatic niche in the liver is more readily established. In this context, CXCR2 inhibition may confer multiple benefits. As the recruitment of granulocytic MDSCs decreases, T cells may more readily enter tumor tissues. *MMP-9*- and *VEGF*-associated vascular abnormalities may also be alleviated (Nie et al., 2024). The supportive effects of myeloid cells on metastatic colonization may likewise be weakened. Clinical evidence further supports this view, as high CXCL8/interleukin-8 (IL-8) expression is associated with CRC invasion, metastasis, and poor prognosis (Ning et al., 2011). In addition, it can enhance colon cancer cell migration, angiogenesis, and therapeutic tolerance. Accordingly, CXCL8-CXCR2 signaling may serve as an important basis for identifying patients who are suitable for CXCR2-targeted therapy.

However, CXCR2 inhibitors should not be used as non-selective monotherapy. Instead, they should be incorporated into a combination treatment framework for CRC. In a subset of patients with MSS-CRC, CXCR2 blockade may have greater value for immune remodeling. These patients exhibit increased peripheral low-density neutrophils and enrichment of intratumoral *CD66b* ^+^CXCR2^+^ cells (Xu et al., 2025; Zhang et al., 2025b; Køstner et al., 2021; Wang et al., 2023b). Meanwhile, CD8^+^ T cells are predominantly excluded within stromal regions. On the basis of these features, CXCR2 blockade may complement multiple therapeutic modalities. When combined with programmed cell death protein 1/programmed death-ligand 1 (PD-1/PD-L1) antibodies, it may help attenuate myeloid-mediated immune exclusion and enhance T-cell effector function (Hu et al., 2026b; Liao et al., 2019). When combined with bevacizumab, it may simultaneously target aberrant vasculature and myeloid cell recruitment (Rahma and Hodi, 2019). Notably, MDSC recruitment in CRC is characterized by substantial redundancy. Pathways involving CCR2, colony-stimulating factor 1 receptor (CSF1R), C-X-C motif chemokine receptor 4 (CXCR4), and complement component 5a (C5a) may compensate for CXCR2 blockade. Therefore, the CXCL-CXCR2 axis represents one of the MDSC recruitment targets in CRC with a relatively strong mechanistic basis and translational potential.

### 4.2 Targeting *STAT3* signaling

Signal transducer and activator of transcription 3 (*STAT3*) is a central hub linking myeloid-derived suppressor cell (MDSC) expansion to immunosuppressive function. In the CRC microenvironment, factors such as IL-6, interleukin-11 (IL-11), granulocyte-macrophage colony-stimulating factor (GM-CSF), and prostaglandin E2 (PGE2) are often persistently elevated (Xie et al., 2025; Draghiciu et al., 2015). These signals can activate the Janus kinase (JAK)-*STAT3* pathway in myeloid cells, thereby progressively skewing immature myeloid cells toward an immunosuppressive state. After *STAT3* activation, multiple classes of suppression-associated molecules may be upregulated (Liao et al., 2025; Vasquez-Dunddel et al., 2013). This results in sustained arginine depletion and continuous accumulation of ROS and RNS. Consequently, T-cell receptor signaling is impaired, and antigen-presenting capacity is also reduced. Studies using clinical samples and animal models have indicated that increased MDSC abundance is closely associated with CRC progression and suppression of T-cell function (Liu et al., 2026a). In inflammation-associated CRC, the IL-6-*STAT3* pathway may also promote intestinal tumorigenesis and sustain a chronic immunoinflammatory state. Therefore, *STAT3* not only acts on CRC epithelial tumor cells but also participates in shaping a suppressive myeloid ecology.

The core objective of *STAT3* targeting is to remodel the functional state of MDSCs. After direct inhibition of *STAT3*, *ARG1*-, *iNOS*-, and ROS-associated programs in MDSCs are downregulated (Li et al., 2024c; Li et al., 2024b; Vasquez-Dunddel et al., 2013). Their suppressive effects on CD8^+^ T cells and NK cells are also attenuated. Blockade of upstream IL-6/JAK, *VEGF*, or PGE2 signaling may further restrict the expansion and maintenance of MDSCs (Draghiciu et al., 2015). This strategy may be more applicable to patients with CRC characterized by high IL-6 expression, elevated phosphorylated *STAT3* (p*STAT3*), enrichment of *ARG1* ^+^ myeloid cells, and restricted CD8^+^ T-cell proliferation. Compared with CXCR2 blockade, *STAT3* inhibition acts more prominently within the tumor. CXCR2 blockade primarily reduces MDSC recruitment, whereas *STAT3* inhibition weakens the immunosuppressive activity of MDSCs after they enter tumor tissues. The two approaches are therefore complementary. *STAT3* is also involved in intestinal mucosal repair, hematopoietic homeostasis, and anti-infective responses. Long-term, non-selective inhibition may therefore increase the risk of mucosal injury, myelosuppression, or infection (Etwebi et al., 2022; Tang et al., 2024). Accordingly, *STAT3*-directed therapy in CRC should place greater emphasis on short-course intervention, combination application, and myeloid-targeted delivery. *STAT3* targeting may be combined with PD-1 antibodies. This approach may help relieve MDSC-mediated functional suppression before T-cell reactivation (Li et al., 2024b; Tong et al., 2024). It may also be combined with anti-*VEGF* therapy to simultaneously interrupt aberrant vascular and myeloid-supportive signals (Draghiciu et al., 2015). When used sequentially with chemotherapy, it may reduce treatment-induced IL-6/*STAT3*-driven myeloid rebound (Li et al., 2024c). Therefore, *STAT3* targeting should not be separated from patient stratification. Only when *STAT3*-dependent, MDSC-dominated immunosuppression is clearly present are such interventions more likely to produce durable clinical benefit.

### 4.3 MDSC depletion and reprogramming

Standard treatments for CRC markedly alter the distribution and functional state of MDSCs. Chemotherapy, anti-angiogenic therapy, and radiotherapy should not be designed solely according to the conventional concept of tumor debulking. Strategies for MDSC depletion or functional reprogramming should be incorporated in parallel. Taking 5-fluorouracil (5-FU) as an example, its effects are not limited to direct tumor cell killing (Yang et al., 2023). 5-FU can selectively induce MDSC apoptosis and restore T-cell-dependent antitumor immunity. This suggests that CRC chemotherapy exerts certain immunomodulatory effects and may reduce the burden of myeloid-mediated immunosuppression. However, this effect is not sufficient. 5-FU may also induce inflammatory feedback, thereby promoting immunosuppressive remodeling (Xu et al., 2021). It is therefore more appropriately regarded as an initial platform for both tumor debulking and relief of myeloid-mediated suppression.

In metastatic CRC, the immune effects of FOLFOX (folinic acid, fluorouracil, oxaliplatin) combined with bevacizumab are also closely associated with MDSCs (Limagne et al., 2016). Pretreatment levels of MDSCs and T helper 17 (Th17) cells may, to some extent, reflect patient responses to this regimen. During treatment, changes in the myeloid immune state also have predictive value. The effects of bevacizumab are not limited to inhibition of *VEGF*-mediated angiogenesis (Rahma and Hodi, 2019). It may also reduce *VEGF*-supported MDSC expansion. From this perspective, MDSCs are not only targets of therapeutic intervention but may also serve as reference indicators for identifying patients who are likely to benefit.

**FIGURE 4:**
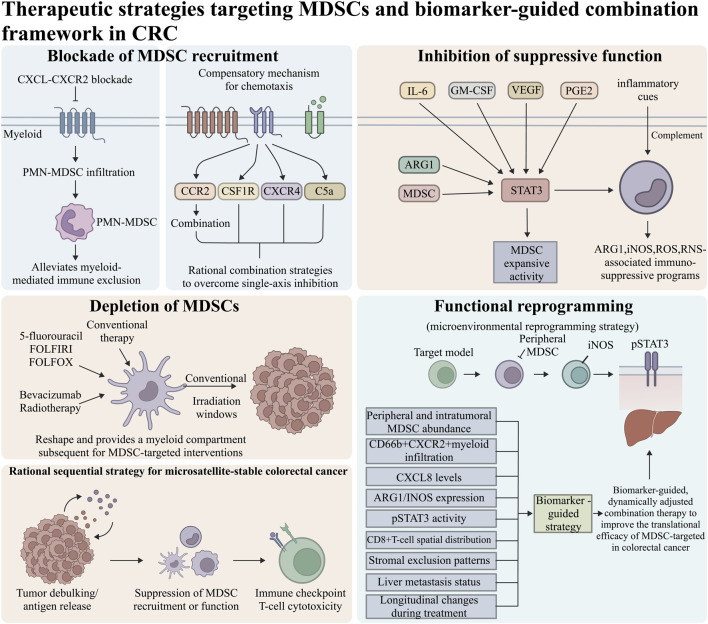
Therapeutic strategies targeting MDSCs and biomarker-guided combination framework in colorectal cancer. MDSC-targeted therapy in colorectal cancer should be positioned as a microenvironmental reprogramming strategy rather than as an isolated monotherapy. Therapeutic approaches can be broadly divided into four categories: blockade of MDSC recruitment, inhibition of suppressive function, depletion of MDSCs, and functional reprogramming. CXCL–CXCR2 blockade may reduce PMN-MDSC infiltration and alleviate myeloid-mediated immune exclusion, whereas CCR2, CSF1R, CXCR4, C5a, and related pathways may compensate for single-axis inhibition and should be considered in rational combination strategies. STAT3 inhibition targets a central signaling hub that links IL-6, GM-CSF, VEGF, PGE2, and inflammatory cues to MDSC expansion and suppressive activity, thereby reducing ARG1, iNOS, ROS, and RNS-associated immunosuppressive programs. Conventional therapies, including 5-fluorouracil, FOLFOX, FOLFIRI, bevacizumab, and radiotherapy, can reshape the myeloid compartment and may provide windows for subsequent MDSC-targeted intervention. In microsatellite-stable colorectal cancer, a rational sequential strategy may involve initial tumor debulking and antigen release, followed by suppression of MDSC recruitment or function, and subsequent immune checkpoint blockade to restore T-cell cytotoxicity. Patient selection should integrate peripheral and intratumoral MDSC abundance, CD66b^+CXCR2^+ myeloid infiltration, CXCL8 levels, ARG1/iNOS expression, pSTAT3 activity, CD8^+^ T-cell spatial distribution, stromal exclusion patterns, liver metastasis status, and longitudinal changes during treatment. Such biomarker-guided, dynamically adjusted combination therapy may improve the translational efficacy of MDSC-targeted approaches in colorectal cancer.

MSS-CRC is typically characterized by pronounced immune exclusion. Reliance on chemotherapy-induced antigen release or radiotherapy-induced inflammation alone is insufficient to fully reverse this state. The underlying mechanisms involve a suppressive barrier jointly formed by multiple cell populations, including MDSCs, cancer-associated fibroblasts (CAFs), and regulatory T cells (Tregs). A more rational strategy is combination therapy rather than simple treatment intensification. FOLFOX, FOLFIRI (folinic acid, fluorouracil, irinotecan), or local radiotherapy may first be used to reduce tumor burden and promote antigen release (Boustani et al., 2024). Inhibitors targeting CXCR2, *STAT3*, *ARG1*, or the adenosine pathway may then be introduced to limit post-treatment MDSC rebound (Bhattacharyya et al., 2025). Finally, PD-1/PD-L1 antibodies may be combined to restore T-cell cytotoxic function. The focus of this strategy is not the simple superimposition of agents but the selection of appropriate intervention windows according to the myeloid dynamics after CRC treatment. In addition to efficacy endpoints, the proportions of peripheral and intratumoral MDSCs, *ARG1*/*iNOS* expression, CXCL8 levels, and the spatial distribution of CD8^+^ T cells should be continuously monitored. These indicators may further inform when MDSCs should be depleted and when their functional state should be reprogrammed. Only in this way may the translational efficiency of immunotherapy for MSS-CRC be improved (Figure 4) (Table2).

**TABLE 2:** Therapeutic strategies targeting MDSCs in colorectal cancer. This table summarizes major MDSC-targeted therapeutic approaches in colorectal cancer, including blockade of MDSC recruitment, inhibition of STAT3-dependent programming, chemotherapy-associated MDSC depletion, anti-angiogenic immune remodeling, reversal of metabolic suppression, combination with immune checkpoint blockade, and spatially guided microenvironmental reprogramming. For each strategy, the table lists the main target or pathway, mechanistic rationale, potentially suitable patient subgroup or biomarker, and key limitations.

Strategy	Main target or pathway	Mechanistic rationale in CRC	Potentially suitable subgroup or biomarker	Key limitations
Blocking MDSC recruitment	CXCL-CXCR2, CCL-CCR2, CXCR4 and C5a axes	Chemokine gradients recruit MDSCs into invasive margins, hypoxic regions, stromal compartments and metastatic niches; CXCR2 is particularly relevant to PMN-MDSC migration	MSS CRC with high CXCL8/IL-8, CD66b^+^CXCR2^+^ cells, low-density neutrophils or liver-metastatic tendency	Redundant chemokine networks may compensate for single-axis blockade
Targeting STAT3-dependent programming	IL-6/JAK/STAT3, IL-11, GM-CSF, PGE2 and VEGF-related signals	STAT3 promotes MDSC expansion and ARG1/iNOS/ROS/RNS-mediated suppression of CD8^+^ T cells and NK cells	CRC with high IL-6, pSTAT3^+^ myeloid cells, ARG1^+^ MDSCs or poor CD8^+^ T-cell proliferation	Non-selective inhibition may affect mucosal repair, hematopoiesis and host defense
Chemotherapy-associated MDSC depletion	5-FU-based chemotherapy, FOLFOX and FOLFIRI	5-FU can induce MDSC apoptosis and partially restore T-cell-dependent antitumor immunity	Patients receiving systemic chemotherapy with monitorable MDSC, ARG1/iNOS or cytokine dynamics	Chemotherapy may trigger inflammatory feedback and myeloid rebound
Anti-angiogenic immune remodeling	VEGF/VEGFR axis and bevacizumab-based regimens	VEGF supports angiogenesis and MDSC expansion; anti-VEGF therapy may reduce vascular abnormality and myeloid suppression	Metastatic CRC with VEGF-high, MDSC-enriched or angiogenesis-dominant features; pretreatment MDSC/Th17 levels may be informative	Immune benefit is context-dependent and usually requires combination therapy
Reversing metabolic suppression	ARG1, iNOS, ROS/RNS, NO and CD39/CD73-adenosine signaling	MDSCs deplete L-arginine and generate ROS/RNS/NO; hypoxia promotes adenosine accumulation, suppressing T and NK cells	Hypoxic, lactate-rich, ARG1/iNOS-high or immune-excluded MSS CRC.	Optimal biomarkers, timing and combinations remain unclear
Combination with immune checkpoint blockade	MDSC-targeted therapy plus PD-1/PD-L1 blockade	Reducing MDSC-mediated exclusion or suppression may render myeloid-dominant CRC more permissive to ICB.	MSS CRC with CD8^+^ T-cell exclusion, high MDSC burden, elevated CXCL8/CXCR2 activity or ICB resistance	Requires rational sequencing; simple drug addition may increase toxicity
Spatially guided microenvironmental reprogramming	MDSCs, CAFs, TAMs, Tregs, abnormal vessels and stromal barriers	MDSCs cooperate with stromal, vascular and suppressive immune cells to maintain immune exclusion	Desmoplastic, CAF/TAM-rich or myeloid-dominant CRC with stromal CD8^+^ T-cell retention	Spatial biomarkers and standardized clinical assays remain insufficient

## 5 Conclusion and future perspectives

MDSCs provide a critical perspective for understanding immune hyporesponsiveness in CRC. Their significance lies not merely in indicating an expansion of myeloid cells, but more importantly in revealing an immunosuppressive state shaped by the concerted effects of multiple factors, including tumor evolution, host inflammation, the gut microbiota, tissue architecture, and therapeutic pressure. In MSS CRC, the limited efficacy of immunotherapy cannot be simply attributed to an insufficient mutational burden. A more fundamental barrier arises from the combined constraints imposed by the suppressive myeloid ecosystem, stromal exclusion, aberrant vasculature, and the microenvironment of metastatic organs. Thus, research on MDSCs provides a framework that more closely reflects the actual course of disease progression for explaining the clinical phenomenon of “immune presence without sufficient effector function.”

This field continues to face substantial translational bottlenecks. A unified definition of human MDSCs remains lacking. Considerable heterogeneity among studies in marker combinations, sample processing, and functional validation has rendered inter-study comparisons difficult. Reliance solely on phenotypic stratification often fails to distinguish truly immunosuppressive pathological myeloid cells from conventional neutrophils or monocytes involved in inflammatory responses. Current evidence also remains largely confined to correlative observations. High-quality studies capable of concurrently integrating function, spatial localization, and clinical outcomes in patient tissues remain insufficient. Conventional animal models likewise have inherent limitations. They are unable to fully recapitulate the mucosal barrier, microbial exposure, gut-liver axis, and long-term treatment-imposed selective pressure observed in human CRC. This, to some extent, constrains the translational value of basic discoveries in clinical settings. In addition, MDSC-associated networks exhibit substantial redundancy. Interventions targeting a single molecule or pathway are therefore readily attenuated by alternative recruitment mechanisms and functional compensation.

Future research should shift from the mere quantification of cell numbers toward the assessment of functional states and their value in clinical decision-making. The identification of MDSCs should not rely on a single parameter. Instead, surface markers, transcriptional profiles, metabolic states, suppressive functions, and spatial relationships should be integrated to establish a reproducible and generalizable standardized evaluation framework. Spatial omics, multiplex imaging, single-cell omics, and artificial intelligence-assisted pathological analysis may help clarify the actual significance of distinct myeloid states. Particular emphasis should be placed on critical regions, including tumor nests, the invasive front, perivascular areas, and metastatic lesions. In parallel, patient-derived organoids, tumor slice cultures, humanized immune models, and gut-on-a-chip technologies require further integration. These platforms should be combined with longitudinal follow-up of clinical specimens to improve the capacity of experimental systems to recapitulate the real trajectory of disease progression.

MDSC-targeted therapy should not be regarded as an isolated monotherapeutic strategy. A more appropriate positioning is as a component of microenvironmental reprogramming intended to enhance the accessibility of immunotherapy. Future clinical trials require more refined patient stratification. Stratification criteria should include peripheral myeloid lineages, the spatial immune architecture of tumors, levels of inflammatory mediators, and dynamic changes after treatment. On this basis, it may be possible to determine which patients are more suitable for MDSC depletion and which patients may benefit more from blockade of recruitment or functional reprogramming. For immune-excluded MSS CRC, a more promising direction is not the simple addition of multiple agents, but the sequential integration of chemotherapy, anti-angiogenic therapy, myeloid-targeted intervention, and ICB. Only through the coordinated advancement of standardized definitions, faithful disease models, dynamic monitoring, and biomarker-driven trials can MDSC research be truly translated into a clinical tool for improving patient prognosis.
